# The association between relapse and the outcome of schizophrenia and recurrent psychotic disorders

**DOI:** 10.1192/bjp.2024.304

**Published:** 2025-10

**Authors:** Joanna Moncrieff, Elizabeth Pillai, Louise Marston, Glyn Lewis, Thomas R. E. Barnes, Sonia Johnson, Stefan Priebe

**Affiliations:** Division of Psychiatry, University College London, UK; North East London Foundation Trust, London, UK; Research Department of Primary Care and Population Health, University College London, UK; Division of Psychiatry, Imperial College London, UK; Centre for Mental Health Research, City St George’s, University of London, UK

**Keywords:** Antipsychotics, employment, psychotic disorders/schizophrenia, relapse, social functioning

## Abstract

**Background:**

Having a relapse of schizophrenia or recurrent psychosis is feared by patients, can cause social and personal disruption and has been suggested to cause long-term deterioration, possibly because of a toxic biological process.

**Aims:**

To assess whether relapse affected the social and clinical outcomes of people enrolled in a 24-month randomised controlled trial of antipsychotic medication dose reduction versus maintenance treatment.

**Methods:**

The trial involved participants with a diagnosis of schizophrenia or recurrent, non-affective psychosis. Relapse was defined as admission to hospital or significant deterioration (assessed by a blinded end-point committee). We analysed the relationship between relapse during the trial and social functioning, quality of life, symptom scores (Positive and Negative Syndrome Scale) and rates of being in employment, education or training at 24-month follow-up. We also analysed changes in these measures during the trial among those who relapsed and those who did not. Sensitivity analyses were conducted examining the effects of ‘severe’ relapse (i.e. admission to hospital).

**Results:**

During the course of the trial, 82 out of 253 participants relapsed. There was no evidence for a difference between those who relapsed and those who did not on changes in social functioning, quality of life, symptom scores or overall employment rates between baseline and 24-month follow-up. Those who relapsed showed no change in their social functioning or quality of life, and a slight improvement in symptoms compared to baseline. They were more likely than those who did not relapse to have had a change in their employment status (mostly moving out of employment, education or training), although numbers changing status were small. Sensitivity analyses showed the same results for those who experienced a ‘severe’ relapse.

**Conclusions:**

Our data provide little evidence that relapse has a detrimental effect in the long term in people with schizophrenia and recurrent psychosis.

Schizophrenia and related psychotic conditions affect 0.2–0.3% of the global population^1^ and cause considerable disability.^2^ They have a particularly detrimental impact on social and occupational functioning,^3,4^ which contributes to the high economic costs of the conditions.^5,6^ There is significant heterogeneity in the trajectory of the conditions, but for many it follows a relapsing–remitting course. Relapses cause social and psychological disruption to patients’ lives and are a substantial source of concern for patients.^7^ They place a burden on caregivers^8^ and health services.^9^ It has also been suggested that acute psychotic relapse may be associated with biological harm^10,11^ that drives progression of the illness,^12^ and reduces treatment responsiveness.^13,14^ For some commentators, the relationship between relapse and prognosis is thought to occur primarily during a ‘critical period’ early in the course of the condition, during which there is a potentially reversible decline, followed by a ‘plateau’.^11,15^ Others suggest the relationship between relapse and prognosis is general and ongoing.^12,14^

## Existing evidence

The evidence on whether relapse affects outcomes is mixed. Clinically, there is an association between relapse and poor psychosocial outcomes and non-remission^16,17^ and some studies have shown that people with a first episode of psychosis become less responsive to antipsychotic treatment following relapse,^18,19^ but these findings are potentially confounded by the underlying severity of the illness. Most longitudinal studies find that symptoms revert to pre-relapse levels^20–22^ and one study found few differences in clinical and quality-of-life outcomes between individuals who relapsed and those who did not over a 6-month period.^23^ However, a 10-year follow-up of a placebo-controlled trial of antipsychotic maintenance in people with a first episode of psychosis found that relapse mediated a poorer long-term outcome in people originally randomised to antipsychotic discontinuation.^24^ Whether relapse impacts the outcomes of schizophrenia and related disorders, and if so how, is highly relevant for patients and clinicians and is likely to have an influence on decisions about treatment, given that maintenance antipsychotic treatment is associated with lower rates of relapse.^25,26^ Further data are required, especially from robust studies, that address how relapse affects clinical and social outcomes. The current study addresses the effects of relapse in a cohort of people with long-term conditions.

## Method

The current paper presents a secondary analysis of data from a 24-month randomised trial that compared maintenance antipsychotic treatment with supported antipsychotic reduction. The present analysis treats participants recruited to the trial as a single cohort. The aim was to compare the outcomes of participants who relapsed and those who did not relapse during the course of the trial. The outcomes we examined were social functioning, quality of life, symptom scores and employment. We also assessed changes in outcomes from baseline to final follow-up in participants who relapsed and those who did not.

The trial, known as the RADAR (Research into Antipsychotic Discontinuation and Reduction) trial was an open, parallel-group, randomised trial that ran between 2017 and 2022. It compared the outcomes of antipsychotic maintenance treatment with a gradual and flexible reduction and discontinuation regimen. The primary outcome was social functioning at the 24-month follow-up, while secondary outcomes included relapse, psychiatric symptoms, quality of life, side effects and neuropsychological performance.

The authors assert that all procedures contributing to this work comply with the ethical standards of the relevant national and institutional committees on human experimentation and with the Helsinki Declaration (https://www.wma.net/policies-post/wma-declaration-of-helsinki-ethical-principles-for-medical-research-involving-human-subjects/) of 1975, as revised in 2013. All procedures involving human participants/patients were approved by Brent Research Ethics Committee (reference16/LO/1507). The trial was registered with the ISRCTN registry (ISRCTN90298520) and with ClinicalTrials.gov (http://clinicaltrials.gov/) (NCT03559426, http://clinicaltrials.gov/show/NCT03559426).

The trial methods have been described in detail elsewhere.^27^ Briefly, participants were aged 18 years and above and had a diagnosis of recurrent non-affective psychotic disorder, for which they were prescribed antipsychotic medication. The exclusion criteria included an admission to a psychiatric hospital or a mental health crisis within the preceding month, being mandated to take their medication through the Mental Health Act and being considered to pose a serious risk to themselves or others by their treating clinicians. Written, informed consent was obtained from all participants, who were then randomised to receive either maintenance antipsychotic treatment or an individualised dose reduction regimen, administered with the guidance of their treating clinician.

Research assessments were conducted at 6, 12 and 24 months. Relapse was defined either as admission to hospital or as a psychotic episode involving significant functional deterioration as adjudicated by a blinded, expert, end-point committee. The committee applied pre-defined criteria on the basis of information from clinical records, which was redacted for information that might reveal a participant’s randomised group.

A statistical analysis plan for the current analysis was developed before the analysis. We aimed to explore whether having a relapse during follow-up affected 24-month measures of the Social Functioning Scale (SFS),^28^ the Manchester Short Assessment of Quality of Life (MANSA),^29^ the total score on the Positive and Negative Syndrome Scale (PANSS) (sum of the subscales)^30^ and employment status, categorised as being or not being in employment, education or training.

We combined severe and non-severe relapses for the purposes of this analysis, since we were confident our assessment procedure only identified significant relapse episodes and not minor symptom fluctuations. Sensitivity analyses were conducted to investigate the effects of ‘severe’ relapse (defined as admission to hospital) versus having a non-severe or no relapse to explore the consequences of having a severe relapse compared to not having one. For all analyses, we combined data from both randomised groups, and adjusted for randomised group in the adjusted analysis.

Analyses were conducted using linear regression with robust standard errors for the continuous outcomes and logistic regression with robust standard errors for employment status. Robust standard errors were used because of the potential clustering of data by site. Initially, an analysis was conducted adjusting only for baseline values. The analysis was repeated, adjusting for age and gender in addition to these variables, and then again adjusting additionally for demographic and clinical variables that varied between people who relapsed and those who did not (using an *a priori* threshold of *P* < 0.1) and might credibly have been associated with relapse and outcomes. Since the literature suggests there are no reliably consistent predictors of relapse,^31,32^ we did not adjust for other variables. The distribution of PANSS scores was slightly skewed, so a further sensitivity analysis was performed using the log-transformed PANSS score. A sensitivity analysis using change scores as the dependent variable was also performed.

To explore whether the participants who experienced a relapse returned to baseline levels of functioning, quality of life and symptoms, we performed paired *t*-tests to compare 24-month values of outcome measures to baseline values. We repeated this analysis for those participants who did not relapse, for comparison purposes. We also compared changes in employment, education and training status between those who relapsed and those who did not.

## Results

Two hundred and fifty-three participants were recruited and randomised into the RADAR trial and 190 participants completed the research assessments at 24-month follow-up. The majority of participants were male, White, single and unemployed. Most had been in contact with mental health services for more than 10 years, with about a third for over 20 years (Table 1).

**Table 1:** Baseline characteristics of people who subsequently relapsed and people who did not

Characteristics	Relapsed (*n* = 82)	Not relapsed (*n* = 171)	Test (*t*-test, chi-squared or Mann–Whitney *U*-test, *z*)
Age, mean (s.d.)	44.25 (12.82)	47.30 (11.21)	*t* = 1.93, *df* = 251 (*P* = 0.55)
Gender			
Men	52/82 (63.4%)	116/171 (67.8%)	*X* ^2^ (2, *n* = 253) = 1.883, *P* = 0.390^a^
Women	28/82 (34.1%)	54/171 (31.6%)	
Transgender	2/82 (2.4%)	1/171 (0.6%)	
Marital status			
Single, separated, divorced or widowed	63/82 (76.8%)	153/171 (89.5%)	*X*² (1, *n* = 253) = 7.10, *P* = 0.008
Married, cohabiting or in a civil partnership	19/82 (23.2%)	18/171 (10.5%)	
Ethnicity			
White	57/80 (71.3%)	114/168 (67.9%)	*X*² (3, *n* = 248) = 1.387, *P =* 0.709
Black	14/80 (17.5%)	38/168 (22.6%)	
Asian	5/80 (6.3%)	11/168 (6.5%)	
Other ethnicities	4/80 (5.0%)	5/168 (3.0%)	
Years of completed education, mean (s.d.)	13.43 (2.91)	14.05 (3.86)	*t* = 1.403, *df* = 190.87 (*P* = 0.162)
Employment			
In employment, education or training	21/80 (25.3%)	53/171 (31.0%)	*X*² (1, *n* = 251) = 0.590, *P* = 0.442
Not in employment, education or training	59/80 (73.8%)	118/171 (69.0%)	
Diagnosis			
Schizophrenia	51/82 (62.2%)	123/171 (71.9%)	*X*² (1, *n* = 253) = 2.45, *P* = 0.118
Other psychotic disorders	31/82 (37.8%)	48/171 (28.1%)	
Duration of contact with mental health services			
0–3 years	4/82 (4.9%)	13/171 (7.6%)	*X*² (5, *n* = 253) = 1.526, *P* = 0.910
4–10 years	20/82 (24.4%)	42/171 (24.6%)	
11–15 years	13/82 (15.9%)	30/171 (17.5%)	
16–20 years	13/82 (15.9%)	29/171 (17.0%)	
>20 years	32/82 (39.0%)	57/171 (33.3%)	
Number of previous mental health admissions median (interquartile range)	3 (1–5)	3 (1–5)	*z* = −1.295 (*P* = 0.195)
Recreational drugs used over the past month	9/82 (11.0%)	16/170 (9.4%)	*X*² (1, *n* = 252) = 151, *P* = 0.697
Alcohol use over past month			
1 × a month or less	57/82 (69.6%)	105/170 (61.8%)	*X*² (2, *n* = 252) = 1.46, *P* = 0.483
2–4 × a month	12/82 (14.6%)	32/170 (18.8%)	
2+ a week	13/82 (15.9%)	33/170 (19.4%)	
Dose of antipsychotic medication (in chlorpromazine equivalents in milligrams), mean (s.d.)	351.05 mg (224.38 mg)	370.87 mg (265.85 mg)	*t* = −0.583, *df* = 251 (*P* = 0.561)
Digit span	14.27 (4.31)	14.94 (4.68)	*t* = 1.09, *df* = 248 (*P* = 0.279)
Randomised group			
Antipsychotic dose reduction	54/82 (65.9%)	72/171 (42.1%)	*X*² (1, *n* = 253) = 12.50, *P* < 0.001
Antipsychotic maintenance medication	28/82 (34.1%)	99/171 (57.9%)	
SFS baseline, mean (s.d.)	107.67 (9.15)	108.07 (9.54)	*t* = −0.317, *df* = 241 (*P* = 0.751)
MANSA baseline, mean (s.d.)	4.76 (0.81)	4.60 (0.82)	*t* = 1.495, *df* = 251 (*P* = 0.136)
PANSS baseline, mean (s.d.)	49.50 (15.01)	52.78 (15.85)	*t* = −1.546, df = 243 (*P* = 0.123)

SFS, Social Functioning Scale; MANSA, Manchester Short Assessment of Quality of Life; PANSS, Positive and Negative Syndrome Scale total score.

a . Chi-squared test for men and women only was *X*² (1, *n* = 250) = 0.258, *P* = 0.611.

The proportion of participants who completed the SFS at follow-up was slightly lower among those who had relapsed (67.1%) than those who had not relapsed (74.5%). For the MANSA, the proportions were 64.6% for those who had relapsed versus 71.4% for those who had not and, for the PANSS, the proportions were 42.7 and 44.4%, respectively. Employment, education and training status was available for 70.7% of the participants who had relapsed and 77.2% of those who had not. None of these differences were statistically significant (see Supplementary Table S1, available at https://doi.org/10.1192/bjp.2024.304).

The results of the trial have been published elsewhere.^33^ Altogether, 82 participants had at least one relapse of any severity while 171 did not experience any relapse. There were few differences between those who relapsed and those who did not on demographic or clinical variables at baseline. The participants who relapsed were more likely to have been randomised to the antipsychotic dose reduction group (Table 1) and to be married or in a long-term partnership. There was no evidence of any differences in social functioning, quality of life or symptoms.

Table 2 shows the 24-month outcome results for the participants who relapsed and those who did not. Controlling for baseline values alone showed no statistically significant differences on the measures of social functioning, quality of life, symptoms or rates of being in employment, education or training; adjusting for age and gender did not alter this result. Adjusting for the randomised group and marital status in addition to age and gender did not change the direction or statistical significance of any results.

**Table 2:** Regression analysis of relationships between outcomes and occurrence of relapse

Outcome	24-month outcome mean (s.d., *N*)		Coefficient for association of relapse and outcome (95% CI) adjusted for baseline values	Coefficient (95% CI) adjusted for age and gender	Coefficient (95% CI) adjusted for age, gender, randomised group and marital status
	Relapsed	Not relapsed			
SFS	107.04 (10.11, *n* = 55)	105.88 (10.15, *n* = 129)	0.79 (−1.74, 3.32)	−0.14 (−2.78, 2.51)	−0.63 (−3.38, 2.13)
MANSA	4.61 (0.85, *n* = 53)	4.68 (0.83, *n* = 122)	−0.14 (−0.36, 0.08)	−0.12 (−0.33, 0.09)	−0.12 (−0.34, 0.11)
PANSS	46.91 (13.41, *n* = 35)	49.16 (14.17, *n* = 76)	−1.17 (−5.08, 2.74)	−1.72 (−6.08, 2.64)	−1.17 (−5.77, 3.43)^b^
Rate of employment, education and training	8/66 (12.1%)	30/124 (24.2%)	Odds ratio 1.03 (0.38, 2.80)	Odds ratio^a^ 1.08 (0.41, 2.85)	

SFS, Social Functioning Scale; MANSA, Manchester Short Assessment of Quality of Life; PANSS, Positive and Negative Syndrome Scale total score.

a . This analysis is only adjusted for age and baseline employment status as it was not possible to adjust for gender because of the sample size.

b . Not enough events in the outcome to perform this analysis.

Sensitivity analyses also showed no statistically significant differences on any of the measures between the participants who had a ‘severe’ relapse (admission to hospital) and those who had a non-severe or no relapse (Supplementary Table S2). Using a log-transformed PANSS variable (which reduced skewness) did not alter the results (Supplementary Table S3). The sensitivity analysis of change scores produced very similar results (Supplementary Tables S4 and S5).

Among those who had relapsed, the paired analysis comparing individuals’ 24-month outcome measures with their baseline scores showed no change in the SFS or the MANSA (Table 3). Symptom scores were slightly improved at follow-up in those who had relapsed as well as those who had not. Participants who had not relapsed showed a small decline in social functioning and a small improvement in quality-of-life scores at 24 months, compared with baseline (Table 3). The results were similar using the ‘severe’ relapse definition, with no statistically significant deteriorations in any measures among those who had relapsed and slight improvements in symptom scores (Supplementary Table S6).

**Table 3:** Paired analysis of changes in outcome measures during the course of the trial

Outcome	Baseline mean (s.d.)	24-month follow-up mean (s.d.)	Mean difference (change) (95% CI)
Relapsed			
SFS (*n* = 54)	107.61 (9.20)	106.90 (10.15)	−0.7 (−3.13, 1.73)
MANSA (*n* = 53)	4.64 (0.82)	4.61 (0.85)	−0.03 (−0.25, 0.18)
PANSS (*n* = 34)	51.82 (17.14)	47.03 (13.58)	−4.76 (−8.36, −1.17)
Not relapsed			
SFS (*n* = 122)	107.19 (9.54)	105.79 (10.31)	−1.40 (−2.61, −0.19)
MANSA (*n* = 122)	4.55 (0.79)	4.68 (0.83)	0.14 (0.02, 0.25)
PANSS (*n* = 75)	53.16 (16.03)	48.95 (14.14)	−4.21 (−7.52, −0.90)

SFS, Social Functioning Scale; MANSA, Manchester Short Assessment of Quality of Life; PANSS, Positive and Negative Syndrome Scale total score.

The proportion of participants who moved out of employment, education or training during the 24 months of the trial was higher among those who relapsed than in those who did not relapse, and so was the proportion who moved into employment, education or training. The overall difference in changes in employment status between those who relapsed and those who did not was statistically significant, although the numbers changing status were small (Table 4). The results were similar for those participants who had a severe relapse (Supplementary Table S7).

**Table 4:** Changes in employment, education and training status during the course of the trial

Status	Relapsed	Not relapsed	Total
Moved out of employment, education or training	13 (22.8%)	12 (9.1%)	25 (13.2%)
Moved into employment, education or training	5 (8.8%)	4 (3.0%)	9 (4.8%)
No change (stayed in or not in employment, education or training)	39 (68.4%)	116 (87.9%)	155 (82.0%)
Total	57	132	189

Fisher’s exact *P* = 0.005.

## Discussion

The notion that a relapse of schizophrenia or other psychotic illness may adversely affect the subsequent course of the condition has been a prevalent one. It has contributed to the rationale for relapse prevention through the use of maintenance antipsychotic medication,^12,14,34^ and may have been a disincentive to supporting patients to discontinue antipsychotic medication.^14^ It is an important clinical issue that is likely to affect both patients’ and clinicians’ attitudes to long-term treatment. Although some research indicates an association between frequent relapse and poorer outcomes,^16^ this cannot be assumed to be a causal connection as both may be manifestations of a more severe condition with a worse prognosis.

Our findings provide little support for the view that relapse results in adverse outcomes, at least among people with long-term conditions, which comprised most of our sample. There was no statistical evidence that social functioning, quality of life and symptoms declined by 24-month follow-up in the participants who relapsed compared with those who did not. Employment rates were lower among those who relapsed at follow-up compared to those who did not relapse, but they were also lower at baseline and there was no evidence of a difference in the baseline-adjusted comparison for rates of employment at follow-up. However, the analysis may have lacked power. While relapse was more common in those people randomised to antipsychotic dose reduction and those who were married, adjusting for these factors did not affect the results. Also, restricting the definition of relapse to those who had a severe relapse only (i.e. were admitted to hospital) did not change the results.

Those participants who relapsed showed no decline in their level of functioning, quality of life or symptoms at follow-up compared with their baseline status. This compares with a slight decline in social functioning scores in the participants who did not relapse and a small improvement in quality of life, although the changes were small, and their clinical significance is uncertain. Symptom scores improved across both groups, but completion rates were lower for the PANSS, and this likely reflects selective completion of this long questionnaire by people who had milder symptoms. In contrast, there was a statistically significant difference in changes in employment status between those who relapsed and those who did not. This principally reflected a larger proportion of people who relapsed moving out of employment, education or training during the course of the trial, although the proportion who moved into employment was also higher and numbers changing employment status in any direction were small. However, this finding may indicate that our analysis of overall employment rates was under-powered to detect a difference.

Our findings are in line with data derived from several other clinical trials in this area. For example, one study found that exacerbations of symptoms in people with established schizophrenia who had been randomised to placebo treatment during trials returned to normal within a few weeks,^22^ while another reported that patients who had relapsed rapidly responded to the reinstatement of antipsychotic medication.^21^ A long-term follow-up of patients from a small placebo-controlled trial of depot fluphenazine found that patients who had been randomised to placebo did not have worse outcomes after 7 years, despite having been considerably more likely to relapse.^20^ An 8-month follow-up of participants with schizophrenia who relapsed while assigned to placebo during the course of a placebo-controlled trial of paliperidone found that symptoms returned to baseline levels and were not different from those who had received continuous antipsychotic medication.^35^ This study also found that a small group of people showed a poor response to treatment reinstatement, which may indicate that relapse can lead to a poorer response to medication in some people with schizophrenia. This is also suggested by the results of a first-episode follow-up study^19^ and a national register-based cohort study.^18^ However, these studies are likely to be confounded by the severity of the underlying illness that is manifested in both relapse and poor prognosis and associated with non-adherence with medication, as shown across medicine.^36^

The findings of a 10-year follow-up of a randomised trial also suggested that relapse could modify outcomes.^24^ This study has been influential in supporting the view that relapse may worsen prognosis^34^ but was subject to several limitations, including the use of a post hoc composite outcome and inclusion of data from participants who dropped out early who were not followed up long term.^37^ Further, no differences were found in individual outcomes at follow-up, such as measures of symptoms, functioning and quality of life, between those randomised to placebo substitution and those randomised to maintenance in the randomised trial, despite a higher rate of relapse among the former.

It remains possible that relapse is detrimental to prognosis early on in the course of the condition specifically, as suggested by the ‘critical periods’ hypothesis.^15^ Since our study mostly involved people with long-term conditions, its results may not be consistent with studies that have assessed the consequences of relapse in people with a first episode. It should also be borne in mind that the evidence for a lack of adverse consequences for an episode of psychotic exacerbation largely derives from studies, such as ours, where relapse was generally treated by continuation or reinstatement of antipsychotic medication.

If relapse does have a lasting impact, it remains unclear what the mechanism might consist of. Although a pathological biological process has been proposed, our findings highlight that the mechanism might also be social, such as through the disruption caused by the loss of employment.

### Strengths and limitations

Our data were derived from a relatively large, randomised trial involving people with recurrent episodes of psychosis or schizophrenia. Since our analysis was planned and conducted after the main trial was designed, we did not conduct formal power calculations for it. However, confidence intervals for the SFS were fairly narrow and excluded a difference of 4 points, which was the clinically meaningful difference used in the power calculation of the original trial.^27^ The confidence interval for the difference in PANSS scores was well below the 10–15 points found to correspond to a minimum level of change on the Clinical Global Impressions scale.^38^ For employment, education and training, however, the confidence interval included an odds ratio of less than half. Although odds ratios are difficult to interpret, this might be considered clinically significant.

Relapse was defined according to stringent criteria applied in a systematic manner. Although the present analysis was not a randomised comparison, confounding by severity may be less likely in a trial cohort compared with naturalistic follow-up studies, for various reasons. These include the exclusion of people with the most unstable trajectories and poor prognoses from trial cohorts, and that stopping medication in a planned way in a trial is different from non-adherence or unplanned discontinuation of medication in a naturalistic setting. The latter is likely to be associated with a poor underlying prognosis,^36^ and hence may be more likely to be associated with relapse.^33^ It is possible that such factors can explain the discrepancy between the results derived from trial cohorts, in which people mostly improve following relapse, at least in the short term, and the findings of naturalistic cohorts or database studies.

There was a loss to follow-up for several assessment measures. For example, between a third and a quarter of the original sample did not complete the primary outcome measure (the SFS) at follow-up. Non-completion was higher for the PANSS since it is a long questionnaire, and hence the improvement in PANSS scores during follow-up that was observed across the participants who had relapsed and those who had not may be partly explained by the selective non-completion by those who were more symptomatic at follow-up. However, there was little difference between the rates of completion for those who had relapsed compared with those who had not. Further, many of our follow-up data were collected during the COVID-19 pandemic, and since social activities were restricted by lockdown measures there may have been a ceiling effect on social functioning scores for some participants.

### Implications for research

Future studies should explore different outcomes to investigate whether particular aspects of social and psychological functioning might be affected by relapse. Adequately powered studies need to be done to confirm whether relapse has a detrimental effect on employment. The mechanisms by which any negative outcomes occur also require further evaluation.

### Implications for practice

Whether relapse leads to a worse prognosis is important information for patients and clinicians. There are many reasons to try to avoid relapse, such as the individual distress it can cause and the disruption to personal and social lives, and our analysis indicates there may be an impact on employment for some people.^12,34^ However, our results suggest that maintenance antipsychotic treatment cannot be justified by the notion that it averts a progressive deterioration in clinical status or social functioning associated with relapse, at least in people with long-term conditions. While avoiding relapse is, nevertheless, a priority for many people,^39,40^ some may opt to accept an increased risk of relapse to reduce the adverse effects and health consequences of long-term antipsychotic treatment. Our data suggest that if people make this choice and then relapse as a consequence, they will subsequently return to their previous level of functioning, although for most people this is likely to involve restarting antipsychotic treatment. However, the psychosocial disruption associated with a relapse, including possible interruption of employment, also needs to inform any such decision.

Overall, our data do not support the notion that a relapse is associated with persistent deterioration in people who have already experienced psychosis or schizophrenia for several years, although employment status may be affected. This information can help to support informed decision-making about the use of long-term antipsychotic treatment, given the risks of relapse associated with reducing and discontinuing such medication.

## Supporting information

Moncrieff et al. supplementary material Moncrieff et al. supplementary material

## Data Availability

The study investigators own and have complete control of the research data. The study protocol has been published. The data that support the findings of this study are available from the corresponding author, J.M., upon reasonable request.

## Supplementary material

The supplementary material is available online at https://doi.org/10.1192/bjp.2024.304.

## Analytic code availability

The analytic code is available on request from the corresponding author J.M. at j.moncrieff@ucl.ac.uk.
