# Supplementary material for: The association between relapse and the outcome of schizophrenia and recurrent psychotic disorders
Source: Br J Psychiatry. 2025 Apr 14;227(4):673–9. doi: 10.1192/bjp.2024.304 (PMC12492064; doi:10.1192/bjp.2024.304)
Supplement: Moncrieff et al. supplementary material [file S0007125024003040sup001.docx]

**The association between relapse and the outcome of schizophrenia and recurrent psychotic disorders.**

**Supplementary material**

**Contents:**

**Table S1 Numbers who completed 24-month outcome measures**

**Table S2 Sensitivity analyses defining relapse as ‘severe’ (admission to hospital)**

**Table S3 Regression analysis using log PANSS score**

**Table S4 Sensitivity analysis of any relapse using change scores as dependent variables**

**Table S5 Sensitivity analysis of ‘severe’ relapse using change scores as dependent variables**

**Table S6 Paired analysis for people who had a ‘severe’ relapse**

**Table S7 Changes in employment, education and training status during the course of the trial for those who had a ‘severe’ relapse versus those who did not**

**Table S1 Numbers who completed 24-month outcome measures**

|  | **Relapsed (any relapse) N=82** | **Not relapsed N=171** | **Chi squared** |
| --- | --- | --- | --- |
|  |  |  |  |
| **SFS** | 55 (67.1%) | 129 (75.4%) | χ^2^ =1.96; p=0.16 |
| **MANSA** | 53 (64.6%) | 122 (71.3) | χ^2^ =1.17; p=0.28 |
| **PANSS** | 35 (42.7%) | 76 (44.4%) | χ^2^ =0.07; p=0.79 |
| **Employment status** | 58 (70.7%) | 132 (77.2%) | χ^2^ = 1.24; p=0.27 |

**Table S2 Sensitivity analyses defining relapse as ‘severe’ (admission to hospital)**

| **Severe relapse vs non-severe or no relapse** | | | | |  |
| --- | --- | --- | --- | --- | --- |
|  | 24-month outcome | | Unadjusted coefficient (95% confidence interval) | Coefficient (95% confidence interval) adjusted for age and gender | Coefficient (95% confidence interval) adjusted for age, gender, randomised group and marital status |
|  | *Relapsed (severe)* | *No severe relapse* |  |  |  |
| **SFS** | 108.56 (SD 8.99, N=30) | 105.78 (SD 10.30, N=154) | 1.01 (-2.04, 4.06) | -0.18 (-3.24, 2.88) | -0.20 (-3.29, 2.89) |
| **MANSA** | 4.51 (SD 0.91, N=30) | 4.69 (SD 0.81, N=145) | -0.21 (-0.50, 0.07) | -0.23 (-0.51, 0.05) | -0.22 (-0.51, 0.07) |
| **PANSS** | 45.05 (SD 12.51, N=20) | 49.20 (SD 14.15, N=91) | -0.94 (-5.02, 3.13) | -0.88 (-5.07, 3.31) | 0.05 (-4.26, 4.37) |
| **Rate of employment, education and training** | 5/41 (12.2%) | 33/149 (22.1%) | 1.45* (0.36, 5.84) | 1.61*^#^ (0.40, 6.43) | ** |

**Odds ratio, # it was not possible to adjust for gender **there was not enough power to do this analysis*

**Table S3 Regression analysis using log PANSS score (any relapse)**

| 24 month outcome | | Unadjusted coefficient (95% confidence interval) | Coefficient (95% confidence interval) adjusted for age and gender | Coefficient (95% confidence interval) adjusted for age, gender, randomised group and marital status |
| --- | --- | --- | --- | --- |
| *Relapsed* | *Not relapsed* |  |  |  |
| 3.81 (SD 0.27, N=35) | 3.86 (SD 0.28, N=76) | -0.021 (-0.103, 0.062) | -0.033 (-0.124, 0.057 | -0.02 (-0.12, 0.07) |

**Table S4 Sensitivity analysis of any relapse using change scores as dependent variables**

|  | Change scores (mean difference between baseline and 24 month follow-up) (SD, N) | | Unadjusted | Adjusted for age and gender | Adjusted for age, gender, randomised group and marital status |
| --- | --- | --- | --- | --- | --- |
|  | Relapsed | Not relapsed |  |  |  |
| Not controlling for baseline score |  |  | Coefficient (95%CI) | Coefficient (95%CI) | Coefficient (95%CI) |
| SFS change | 0.70 (8.90, N=54) | -1.40 (6.76, N=122) | 0.70 (-1.97, 3.37) | -0.30 (-2.55, 3.16) | -0.14 (-3.12, 2.84) |
| MANSA change | 0.03 (0.77, N=53) | 0.14 (0.64, N=122) | -0.17 (-0.41, 0.07) | -0.13 (-0.36, 0.10) | -0.11 (-0.36, 0.13) |
| PANSS change | -4.76 (10.30, N=34) | -4.21 (14.38, N=75) | -0.55 (-5.35, 4.25) | 1.25 (-6.59, 4.10) | -1.13 (-6.80, 4.54) |
|  |  |  |  |  |  |
| Controlling for baseline score |  |  |  |  |  |
| SFS change | 0.70 (8.90, N=54) | -1.40 (6.76, N=122) | -0.79 (-1.74, 3.32) | -0.14 (-2.78, 2.51) | -0.63 (-3.38, 2.13) |
| MANSA change | 0.03 (0.77, N=53) | 0.14 (0.64, N=122) | -0.14 (-0.36, 0.08) | -0.12 (-0.33, 0.09) | -0.12 (-0.34, 0.11) |
| PANSS change | -4.76 (10.30, N=34) | -4.21 (14.38, N=75) | -1.17 (-5.08, 2.74) | -1.72 (-6.08, 2.64) | -1.17 (-5.77, 3.43) |

**Table S5 Sensitivity analysis of ‘severe’ relapse using change scores as dependent variables**

|  | Change scores (mean difference between baseline and 24 month follow-up) (SD, N) | | Unadjusted | Adjusted for age and gender | Adjusted for age, gender, randomised group and marital status |
| --- | --- | --- | --- | --- | --- |
|  | Relapsed | Not relapsed |  |  |  |
| Not controlling for baseline score |  |  | Coefficient (95%CI) | Coefficient (95%CI) | Coefficient (95%CI) |
| SFS change | -0.81 (8.95, N=30) | -1.26 (7.15, N=146) | 0.45 (-2.95, 3.85) | -0.38 (-3.86, 3.10) | -0.53 (-4.11, 3.05) |
| MANSA change | -0.10 (0.79, N=30) | 0.12 (0.66, N=145) | -0.23 (-0.53, 0.07) | -0.24 (-0.54, 0.06) | -0.23 (-0.54, 0.07) |
| PANSS change | -3.11 (8.63) | -4.66 (13.99, N=90) | 1.55 (-3.30, 6.40) | 1.61 (-3.46, 6.69) | 2.20 (-3.38, 7.77) |
|  |  |  |  |  |  |
| Controlling for baseline score |  |  |  |  |  |
| SFS change | -0.81 (8.95, N=30) | -1.26 (7.15, N=146) | -1.01 (-2.04, 4.06) | -0.18 (-3.24, 2.88) | -0.20 (-3.29, 2.89) |
| MANSA change | -0.10 (0.79, N=30) | 0.12 (0.66, N=145) | -0.21 (-0.50, 0.07) | -0.23 (-0.51, 0.05) | -0.22 (-0.51, 0.07) |
| PANSS change | -3.11 (8.63) | -4.66 (13.99, N=90) | -0.94 (-5.02, 3.13) | -0.88 (-5.07, 3.31) | -0.05 (-4.26, 4.37) |

**Table S6 Paired analysis for people who had a ‘severe’ relapse**

|  | Baseline  Mean (SD) | 24 months  Mean (SD) | Mean difference (change) (95% CI for the difference) |
| --- | --- | --- | --- |
| SFS (N=30) | 109.4 (9.6) | 108.6 (9.0) | -0.81 (-4.15, 2.53) |
| MANSA (N=30) | 4.61 (0.74) | 4.51 (0.91) | -0.10 (-0.40, 0.19) |
| PANSS (N=19) | 48.32 (15.65) | 45.21 (12.83) | -3.11 (-7.27, 1.06) |

**Table S7 Changes in employment during the course of the trial for those who had a ‘severe’ relapse versus those who did had no relapse or a non-severe relapse**

|  | Relapsed (severe) | No severe relapse | Total |
| --- | --- | --- | --- |
| Moved out of employment, education and training | 10 (31.2%) | 15 (9.6%) | 25 (13.2%) |
| Moved into employment, education and training | 4 (12.5%) | 5 (3.2%) | 9 (4.8%) |
| No change (stayed in or not in employment, education or training) | 18 (56.3%) | 137 (87.3%) | 155 (82.0%) |
| Total | 32 | 157 | 189 |

Fisher’s Exact p<0.001
